# Design of Triaxial Tests with Polymer Matrix Composites

**DOI:** 10.3390/polym14040837

**Published:** 2022-02-21

**Authors:** María del Carmen Serna Moreno, Sergio Horta Muñoz, Alberto Ruiz Gracia

**Affiliations:** 1 Instituto de Investigación Aplicada a la Industria Aeronáutica, Escuela de Ingeniería Industrial y Aeroespacial de Toledo, Universidad de Castilla-La Mancha, Av. Carlos III s/n, Real Fábrica de Armas, 45004 Toledo, Spain; sergio.horta@uclm.es; 2 Escuela Técnica Superior de Ingenieros Industriales de Ciudad Real, Universidad de Castilla-La Mancha, Av. Camilo José Cela 2, Edificio Politécnico, 13071 Ciudad Real, Spain; alberto.ruiz6@alu.uclm.es

**Keywords:** triaxial testing, finite element method, polymer-based composites

## Abstract

Multiaxial testing in composites may generate failure modes which are more representative of what occurs in a real structure submitted to complex loading conditions. However, some of its main handicaps include the need for special facilities, the correct design of the experiments, and the challenging interpretation of the results. The framework of this research is based on a triaxial testing machine with six actuators which is able to apply simultaneous and synchronized axial loads in the three space directions. Then, the aim was to design from a numerical point of view a triaxial experiment adapted to this equipment. The methodology proposed could allow for an adequate characterization of the triaxial response of a polymer-based composite with apparent isotropic behaviour in the testing directions. The finite element method (FEM) is applied in order to define the geometry of the triaxial specimen. The design pursues to achieve homogeneous stress and strain states in the triaxially loaded region, which should be accessible for direct measurement of the strains. Moreover, a fixing system is proposed for experimentally reproducing the desired boundary conditions imposed on the numerical simulations. The procedure to determine the full strain tensor in the triaxially loaded region is described analytically and with the help of FEM virtual testing. The hydrostatic component and the deviatoric part of the strain tensor are proposed for estimating the susceptibility of the polymer-based composite to fail due to the triaxial strain state imposed. Then, the loading scenarios that cause higher values of the deviatoric components in the triaxially loaded region are considered to be more prone to damage the region of interest. Nevertheless, the experimental failure is expected to be produced in the arms of the specimen which are uniaxially loaded, since in all of the loading cases the simulations show higher levels of stress concentration out of the triaxially loaded region. Thus, although the triaxial strength could not be accurately determined by the proposed tests, they can be utilized for observing the triaxial response before failure.

## 1. Introduction

The study of stress and strain states due to multiaxial loads has attracted the interest of composite researchers in recent years since they may lead to failure modes more representative of what occurs in a structure which is in service. In particular, biaxial testing [1,2,3,4,5,6,7,8,9,10,11,12,13,14,15,16] could allow studying plane stress conditions that appear frequently in different technological applications in which polymer-based composites are used (for example, in the skin of aerospace vehicles, wind turbines or pressure vessels, which are thin-wall structures and develop mainly biaxial forces contained in their plane). In these cases, if there are areas of angular junction between the different structural parts, additional out-of-plane loads could be non-negligible. However, the analysis of polymer-based composites subjected to triaxial loads is not often investigated in the scientific literature due to its high complexity.

Many of the techniques and equipment used for the study of polymer matrix composites under triaxial loads are based on those developed for the analysis of soils using conventional triaxial tests [17,18,19]. In the case of studying fibre reinforced polymers (FRP), it consists of the use of pressurized cylindrical specimens (in most cases hollowed and with thick walls) to which either axial loads or torsional moments are applied [20,21,22,23,24]. A summary of the main results available in the literature for different materials subjected to a wide range of triaxial loads were compiled by Hinton and Kaddour in [25]. This compilation was made within the framework of the “second world-wide failure exercise” with the intention of evaluating the existing failure models for FRP composite materials under multiaxial load states [26]. The variations found between the theoretical predictions and the experimental results showed that the generation of triaxial stress and strain states experimentally measurable at the laboratory is a pending subject at the research and development level in FRP.

Another different approach, following a philosophy closer to the true triaxial test used in soils, is presented by Welsh and Adams [27]. This trend grows from the need to examine the stiffness and strength of the material submitted to different stress levels in the three directions of the space. This led to the design of specific equipment that allows independent forces to be applied. They designed a triaxial machine that consists of an electromechanical system capable of applying tensile and/or compressive loads on the three orthogonal axes by means of six actuators (two actuators face in each direction). The support frame is the one that ensures the alignment of the six forces. With this equipment, they mainly developed biaxial tests on cross-ply laminates of epoxy matrix reinforced with carbon fibres using cruciform specimens. But subsequently, they proposed using the cross-shaped specimens to perform triaxial tests, applying compressive out-of-plane loads by means of a pair of accessory pieces attached to the actuators that act perpendicularly to the central zone of the specimen [10,27]. However, their cruciform specimens could not be tested under out-of-plane tensile loads.

With the aim of delving into the characteristics and needs of the triaxial test, several studies have extrapolated the geometry of the cruciform specimens for biaxial testing to design a specimen for triaxial testing of isotropic metallic materials. This adaptation indicates that the arms intersection should incorporate new arms that apply perpendicular loads to the gauge region. Cavallaro et al. [28] presented a patent for a triaxial machine in which an adapted specimen was proposed. This specimen is made up of six arms arranged in the three directions of the space, whose ends consist of a larger surface for improving the grip of the jaw. Calloch and Marquis [29] proposed a specimen with a cubic general shape and grooves towards the centre to perform triaxial tests under cyclic loading and unloading. Comanici et al. [30] used a specimen with a similar pattern to the one of Calloch and Marquis to study the stress in the inelastic range under triaxial loads. The specimen of Hayhurst and Felce [31] followed a cruciform geometry in three dimensions, with six arms joined with rounds to the triaxial loaded region. This work is of interest since they measured the change in the dimension of the diagonal of the central region by means of an axial extensometer and the specimen failed in the triaxially loaded region. Nevertheless, as a general outcome, none of the previous specimens consider direct strain measurement in the principal stress directions of the triaxially loaded region.

This work aims to define a methodology that allows for an adequate triaxial characterization of a polymer-based composite with apparent isotropic response in the principal stress directions. Then, with the aim of designing a suitable experiment, the finite element method (FEM) is used to study the stress-strain response of a specimen with a geometry adapted to the triaxial testing installation of the University of Castilla-La Mancha (UCLM, Ciudad Real, Spain), which has similar features to the one designed by Welsh and Adams [27]. The main objective here is to develop a parametric study to find a specimen that permits the direct measurement of the strain state at the triaxially loaded region during the tests. Then, a fixture is designed to accurately reproduce experimentally the desired boundary conditions and to ensure the correct alignment and load transmission to the specimen. As well, homogeneous stress and strain fields are sought in the gauge zone with a reduced level of unavoidable stress concentrations out of the region of interest. Finally, the analytical work needed to estimate the strain tensor from the experiments is presented for different loading combinations.

## 2. Materials and Methods

Numerical simulations are proposed to analyse the stress-strain response of two types of specimen geometries considering an isotropic material. An ad-hoc fixture is defined for imposing the desired boundary conditions during testing. As well, the analytical postprocess of the strain measurements is described in order to obtain the full strain tensor from the experiments.

### 2.1. Material, Equipment and Geometry of the Specimens

This work is focused on the multiaxial response of polymers reinforced with short fibres, the main requirement being that they present an apparent isotropic response in the loading directions. Conventional fabrication processes such as computer numerical control (CNC) machining or injection moulding are possible, although in case of designing complex geometries the option of 3D printing at 45° of the loading directions with a posterior thermal treatment [32,33] could be also advisable. In this study, a chopped glass-reinforced polyester reinforced by 20% of volume is chosen, since in previous research [12,13,14,15] it has been successfully biaxially tested. The average values obtained are E = 6.5 GPa for the Young modulus and *ν* = 0.37 for the Poisson coefficient. It exhibits a brittle behaviour with a mean ultimate stress of *σ_u_* = 90 MPa and strain *ε_u_* = 0.0138.

**Figure 1:**
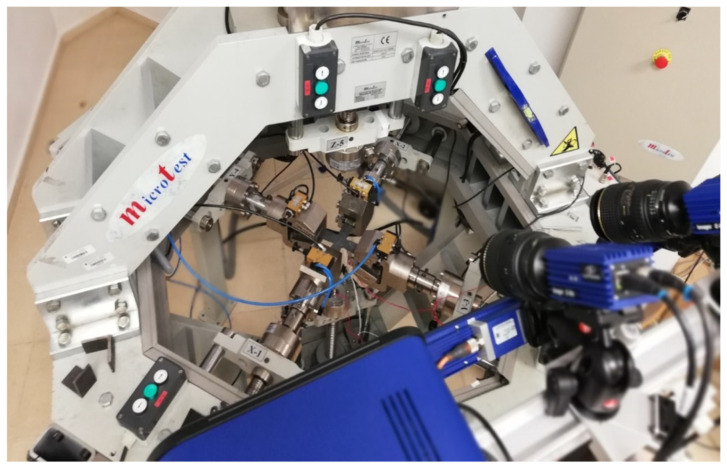
Triaxial testing facility of the UCLM.

The triaxial facility is located in the UCLM and consists in an electromechanical testing machine that allows carrying out tensile and compressive tests in the three space directions by means of six electromechanical actuators (Figure 1). A maximum load of ±50 kN/actuator and a maximum displacement of ±50 mm/actuator can be applied. The pneumatic grips in charge of applying the tensile loads have maximum gripping cross-sectional dimensions of 40 mm × 11 mm.

**Figure 2:**
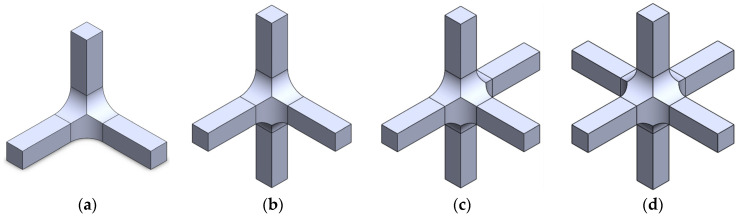
Preliminary design of geometry A: (**a**) CCC loading case; (**b**) CTC loading case; (**c**) CTT loading case; and (**d**) TTT loading case.

**Figure 3:**
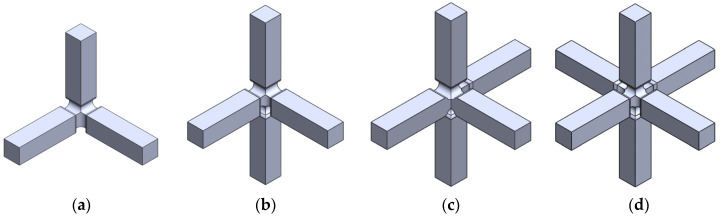
Preliminary design of geometry B: (**a**) CCC loading case; (**b**) CTC loading case; (**c**) CTT loading case; and (**d**) TTT loading case.

**Figure 4:**
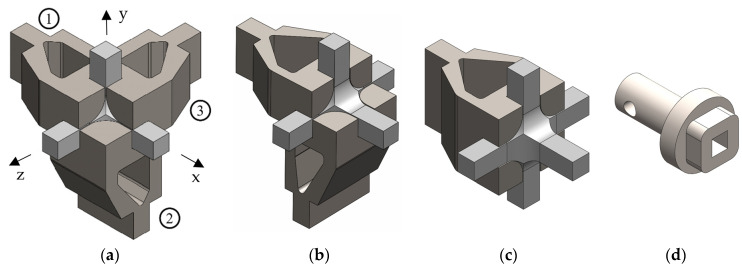
Supporting fixture of the specimen applied to geometry A. The fixture consists of three supporting parts which are numbered in (a) for the x-, y- and z-axes with the etiquettes 1, 2, and 3 respectively. (**a**) CCC loading case; (**b**) CCT loading case; (**c**) CTT loading case; and (**d**) compression platens.

According to the restrictions imposed by the UCLM installation, two triaxial cruciform geometries are proposed. Geometry A and geometry B are shown in Figure 2 and Figure 3, respectively, for the four possible loading cases: (a) compression-compression-compression (CCC), (b) compression-tension-compression (CTC), (c) compression-tension-tension (CTT), and (d) tension-tension-tension (TTT). The specimens seek to leave free zones for measuring the strains in the triaxially loaded region, avoiding as far as possible the full geometry with six arms (Figure 2d and Figure 3d). It is decided that the axes loaded under tension are to have two arms for avoiding undesirable flexion during loading. Meanwhile, the compressed directions consist of a single arm and the fixing system is designed to restrain the displacements in the opposite perpendicular plane (Figure 4). The baseline specimens for the FEM parametric study have an arm length of 40 mm and a squared cross-section of 10 mm side. Therefore, the central cubic region submitted to triaxial loading has the three dimensions of 10 mm. In geometry A, the arms are connected to the gauge region by means of a simple round, while in geometry B a double fillet radius is introduced.

### 2.2. Supporting Fixture

The clamping system is designed to keep the specimen in a fixed position during the test and to assure the alignment of the sample with the actuators under compression. The fixture consists of three supporting parts which, in Figure 4a, are numbered for the x-, y- and z-axes with the labels 1, 2, and 3 respectively. They impose the different boundary conditions for each loading case, requiring one constraining piece for each compressed arm. Then, if the three directions are submitted to compression, the three parts of the fixture have to be used (Figure 4a). Meanwhile, the CTT and CCT loading cases require one and two supporting parts, respectively (Figure 4b,c). No fixture is needed if the six arms are under tension, in which a pneumatic gripping of the specimen is proposed. The compressive loads are conceived to be applied by means of compression plates. To ensure that the arms subjected to compression do not buckle, external walls have been added to the proposed compression platen (Figure 4d). The fixture system is planned to be manufactured in a three-and-a-half-axis milling machine. The material is intended to be a 304 stainless steel, since it is feasible for CNC machining and presents a stiffness of a higher order of magnitude than the composites to be tested.

### 2.3. Determination of the Triaxial Strain State

**Figure 5:**
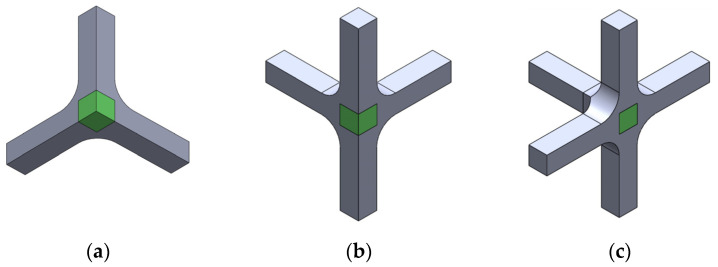
Free surfaces for strain measurement in geometry A: (**a**) CCC loading case; (**b**) CTC loading case; and (**c**) CTT loading case.

There are three measurement methods that have been taken into consideration for directly acquiring the deformations in the triaxially loaded region of the specimen. These are the employment of glued strain gauges, embedded fibre optical sensors, or full field digital image correlation (DIC) techniques. The utilization of inserted sensors could complicate the fabrication process and the DIC system requires to visualise a zone big enough for the correct measurements or the design of a transparent fixture. Therefore, as a first approximation, the use of strain rosette gauges is chosen due to their simple use and reduced size. In particular, the design is developed considering the strain rosette KFGS-1-120-D17-23 T-F7 from Kyowa Electronic Components Co. [34]. It consists of three strain gauges, each with a length of 3 mm, which are positioned at 0°, 90°, and 45°, respectively, on a rosette with a diameter of 5 mm. The number of strain rosettes proposed to carry out the measurements depends on the number of free surfaces in the triaxially loaded region. Therefore, three strain rosettes are proposed to be used in the CCC loading case (Figure 5a), while two and one faces are available under CTC (Figure 5b) and CTT (Figure 5c) loading, respectively.

**Figure 6:**
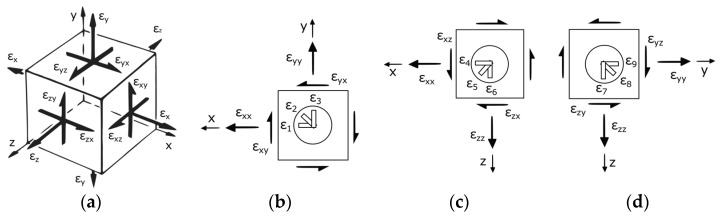
(**a**) Triaxial strain state; (**b**) strain gauge rosette in plane x-y; (**c**) strain gauge rosette in plane x-z; and (**d**) strain gauge rosette in plane y-z.

The aim here is to determine the full strain tensor **ε** given by Equation (1) during testing, whose components are represented in Figure 6a. The hydrostatic part of the strain tensor can be calculated as $\varepsilon_{h}=pI$, I being the 3 × 3 identity matrix and p the hydrostatic component that is obtained from the trace as $p=\left(\varepsilon_{xx}+\varepsilon_{yy}+\varepsilon_{zz}\right)/3$. Then, the deviatoric part of the strain tensor can be expressed as $\varepsilon_{d}=\varepsilon -\varepsilon_{h}$. Figure 6b–d depict a schematic representation of the strain rosette in the x-y, x-z, and y-z planes, respectively. The normal strain components in each plane can be directly measured with the strain gauges (Equation (2)) and the shear components should be calculated according to Equation (3) by combining the direct measurements. Please notice that the elongation of the gauge causes positive strains and its shortening corresponds to negative strains. This sign must be taken into account in Equations (2) and (3).

(1) $$\varepsilon =\left[\begin{array}{ccc} \varepsilon_{xx} & \varepsilon_{xy} & \varepsilon_{xz}\\ \varepsilon_{xy} & \varepsilon_{yy} & \varepsilon_{yz}\\ \varepsilon_{xz} & \varepsilon_{yz} & \varepsilon_{zz} \end{array}\right]$$

(2) $$\begin{array}{c} \varepsilon_{xx}=\varepsilon_{1}=\varepsilon_{4}\\ \varepsilon_{yy}=\varepsilon_{3}=\varepsilon_{9}\\ \varepsilon_{zz}=\varepsilon_{6}=\varepsilon_{7} \end{array}\;$$

(3) $$\begin{array}{c} \varepsilon_{xy}=\varepsilon_{2}-\left(\varepsilon_{xx}+\varepsilon_{yy}\right)/2\\ \varepsilon_{xz}=\varepsilon_{5}-\left(\varepsilon_{xx}+\varepsilon_{zz}\right)/2\\ \varepsilon_{yz}=\varepsilon_{8}-\left(\varepsilon_{yy}+\varepsilon_{zz}\right)/2 \end{array}\;$$

The CCC loading case allows measuring the strain state in three orthogonal planes of the triaxially loaded region, so all of the components of the strain tensor could be obtained during testing. If a CCT loading case is proposed, direct measurements are not possible in the x-y plane and the shear component $\varepsilon_{xy}$ could not be determined. Taking into account the isotropic material response and the hypothesis that the central strains are measured in the principal stress directions, it could be assumed that the shear strain $\varepsilon_{xy}$ (and the other shear strain components) should be negligible compared to the normal strain values. This assumption is verified numerically in Section 4. As well, if a CTT loading case is tested, only a strain rosette could be glued in the y-z plane. Then, not only the shear strain components $\varepsilon_{xy}$ and $\varepsilon_{xz}$ but also the normal strain $\varepsilon_{xx}$ cannot be estimated. If the shear strains are considered to be negligible, the missing normal component could be assessed with a semi-numerical approximation. The FEM allows for obtaining the stress factors $c_{x}$, $c_{y}$, and $c_{z}$ as the relation of the applied stresses $\sigma_{ax}$, $\sigma_{ay}$, and $\sigma_{az}$ with the central stresses $\sigma_{xx}$, $\sigma_{yy}$, and $\sigma_{zz}$. In particular, the relation in the x-direction is $\sigma_{ax}=c_{x}\sigma_{xx}$. Then, according to the generalized Hooke’s law [35], the isotropic material behaviour and the negligible shear terms, the normal strain ε_xx_ can be calculated applying Equation (4). The determination of the correspondent triaxial stress state under TTT loading could be determined from the applied stresses following the semi-numerical approach or by means of direct strain measurements using embedded optical sensors.

(4) $${Ε}_{xx}=\frac{\sigma_{ax}}{Ec_{x}}-\nu \left(\varepsilon_{yy}+\varepsilon_{zz}\right)$$

### 2.4. Numerical Model

Linear static numerical analyses are performed by means of the FEM commercial software ABAQUS^TM^ [36], so non-linearities or buckling are not considered in the simulations. Python scripting is used for parametrizing the definition of the geometry, the boundary conditions, and the generation of the mesh. Regarding the boundary conditions, two different scenarios are modelled. On the one side, the ideal symmetric boundary conditions in the planes of geometrical symmetry are taken into account. On the other side, the displacement constrains imposed by the supporting fixture are considered. These movement restrictions consist of preventing the perpendicular displacements to the surfaces of the specimen that are in contact with the supporting fixture. The 10-node quadratic tetrahedron C3D10 is utilised, that is able to adapt to the complex shaped zones of both geometries A and B. It is a general-purpose element with three translational degrees of freedom per node. A sensitivity analysis to the size of the element is developed until a reasonable level of convergence in the maximum principal stress in the gauge region. Then, an average global size element of 1 mm is used, refining the dimension to 0.5 mm at the fillet radii.

## 3. Results

### 3.1. Design of the Specimen: Influence of the Fillet Radii

**Figure 7:**
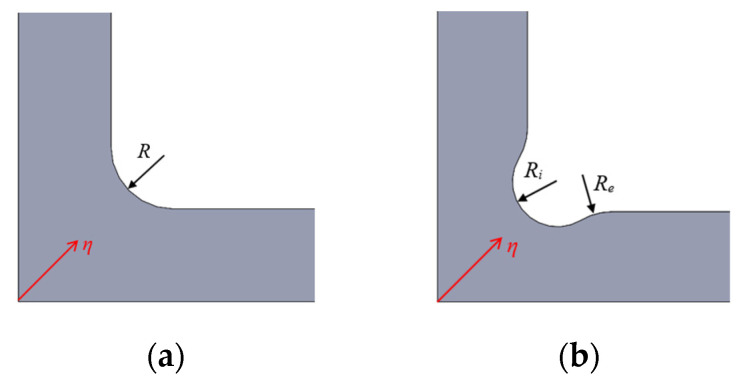
Fillet radii and stress paths in (**a**) geometry A and (**b**) geometry B.

A numerical parametric study of the influence of the fillet radii in the stress and strain states of the specimens under loading is presented. Geometry A has one fillet radius named *R* (Figure 7a), while in geometry B there is an internal radius *R_i_* and an external radius *R_e_* (Figure 7b). A diagonal path η starting in the centre of the specimen (origin of coordinates) is defined in both geometries. The aim is to find the best values in order to achieve a uniform strain state in the triaxially loaded region; the stress concentration out of the region of interest should be as low as possible. This first analysis is carried out in geometries A and B submitted to a CCC loading case with a level of applied compressive stress of 10 kPa. The ideal symmetric boundary conditions in the planes of geometrical symmetry are considered.

#### 3.1.1. Influence of the Fillet Radius in Geometry A

**Figure 8:**
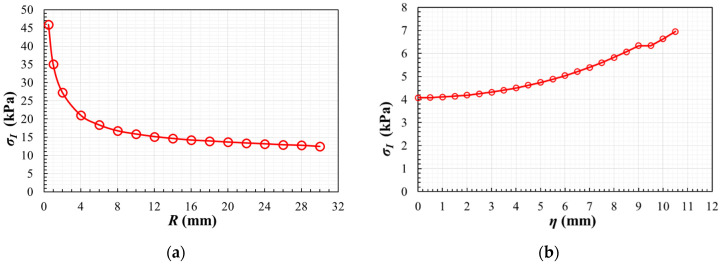
In geometry A: (**a**) the highest value of the maximum principal stress (expressed in absolute value) as a function of the fillet radius; (**b**) maximum principal stress along the path for a fillet radius *R* = 10 mm.

Focusing on the maximum principal stresses *σ_I_*, the highest value in the whole specimen (expressed in absolute value) is depicted in Figure 8a as a function of the fillet radius *R*. The highest maximum principal stresses are obtained out of the gauge region, and they clearly increase with the reduction of the fillet radius. But the highest values present a decrease gradient apparently stable if *R* ≥ 10 mm. This should be considered for deciding which is the chosen fillet radius to achieve low stress concentrations out of the region of interest. The aim here is to delay the undesirable failure and to increase the loading range in which the triaxial response is observable in the centre of the specimen.

Another of the main objectives here is to design a specimen in which the gauge region develops relatively homogeneous stress and strain fields in order to facilitate the experimental measurements and the estimation of the strain tensor. Then, a parametric study using ABAQUS scripting is performed to analyse the homogeneity of the maximum principal stresses along the path η (Figure 7a) for different fillet radii varying between 0.5 mm ≤ *R* ≤ 28.0 mm. In order to exemplify the results, Figure 8b depicts the evolution of the maximum principal stresses along the path for the case with a fillet radius *R* = 10 mm. For the rest of the cases Table 1 lists the position along the path in which there is a variation of 5% and 10% from the maximum principal stress *σ*_0_ at a material point in the origin of coordinates. In particular, Table 1 specifies the η-coordinate that delimits the zone in which the stress fields are considered as homogeneous, since the stresses vary from the central value less than 5% or 10%. As a direct outcome of the analysis, it can be inferred that the increase of the fillet radius reduces the homogeneity of the maximum principal stresses along the path.

The results shown in Figure 8a indicate that an increase in the fillet radius is beneficial for reducing the maximum principal stress outside the measurement zone. However, the results of Table 1 indicate that an increase in the fillet radius causes a decrease in the homogeneity of the maximum principal stresses in the area of interest. As a compromise between the two effects, here the study with geometry A is performed with the intermediate radius of 10 mm. Thus, the stress concentration out of the triaxially loaded zone is not critical and the 3 mm strain gauges that compose the rosette can be placed within a region in which the maximum principal stresses vary less than 10% of the central value.

#### 3.1.2. Influence of the Fillet Radii in Geometry B

**Figure 9:**
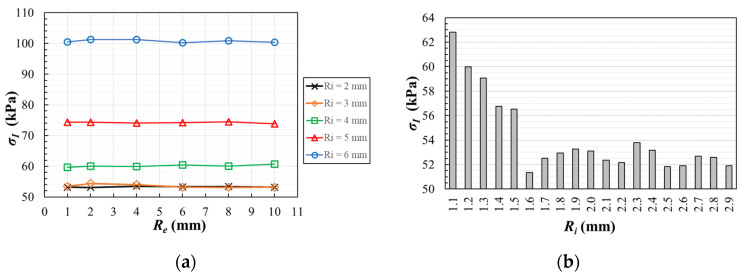
In geometry B: (**a**) The highest value of the maximum principal stress (expressed in absolute value) as a function of the fillet radii. (**b**) For an external radius *R_e_* = 1 mm, influence of the internal radius on the maximum principal stress.

Geometry B demands a parametrization of both the internal and external radii that connect the arms and the gauge region (Figure 7b). As first step, these radii are varied between 1.0 mm ≤ *R_e_* ≤ 10.0 mm and 2.0 mm ≤ *R_i_* ≤ 6.0 mm to find the best combination that reduces as far as possible the unavoidable stress concentration out of the gauge region. Figure 9a depicts the highest maximum principal stresses (in absolute value) as a function of *R_e_* and *R_i_*, observing practically no influence of the external radius in the results. This fact makes possible to eliminate this variable from the analysis due to its little influence, fixing the external radius *R_e_* = 1 mm for the rest of the models. Meanwhile, the highest maximum principal stress decreases when *R_i_* is reduced, obtaining the lowest value with internal radii around 2 mm.

**Figure 10:**
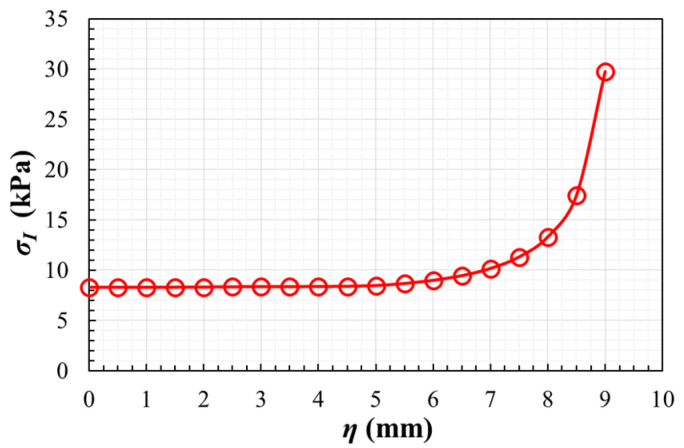
In geometry B, the maximum principal stresses along the path for fillet radii *R_e_* = 1 mm and *R_i_* = 1.6 mm.

Continuing with the parametric study, Figure 9b illustrates the results of a second analysis which is carried out varying the internal radius between 1.1 mm ≤ *R_i_* ≤ 2.9 mm. For an internal radius higher than 1.5 mm, lower stress concentration is found outside of the measurement area. In addition, with the scope of assuring the homogeneity of the stress fields in the gauge region, the maximum principal stresses are calculated along the path η (Figure 7b) for the mentioned interval of *R_i_*. Following the same procedure used in geometry A, Table 2 lists the η-coordinate of the position along the path in which there is a variation of 5% and 10% from the maximum principal stress σ_0_ at a material point in the origin of coordinates. The results show that a larger internal radius causes less homogeneous stress fields. For example, Figure 10 depicts the evolution of the maximum principal stresses along the path for the case with fillet radii *R_e_* = 1 mm and *R_i_* = 1.6 mm. Therefore, for minimizing the stress concentration out of the region of interest and increasing the fields homogeneity in the central zone, the compromise value *R_i_* = 1.6 mm is chosen. Taking into account this decision, it should be noted that *R_e_* = 1 mm is selected since it produces a smoother transition with the arm of the specimen and it facilitates its machining in the case that this is the fabrication method chosen.

#### 3.1.3. Comparison of Geometries A and B

In view of the results of the previous Section 3.1.1 and Section 3.1.2, two of the main differences between the chosen geometries A and B are:

- The highest value of the maximum principal stress (expressed in absolute value) is produced in both cases out of the measurement region, but the lowest value is achieved in geometry A.
- Comparing the maximum principal stress evolution along the paths of geometry A and B, higher homogeneity is found in geometry B.

Keeping as a main aim delaying of the undesirable failure in the central region and increasing the loading range in which the triaxial response is observable in the centre of the specimen, geometry A with a fillet radius of 10 mm would be used as the preferred option. Despite the less homogeneous fields found in geometry A, the rosette allows for the measurement of the strains within a region in which the maximum principal stresses vary less than 10% of the central value. Moreover, geometry A presents a higher geometric simplicity which facilitates its manufacturing, especially in traditional machining and moulding.

### 3.2. Influence of the Supporting Fixture

**Figure 11:**
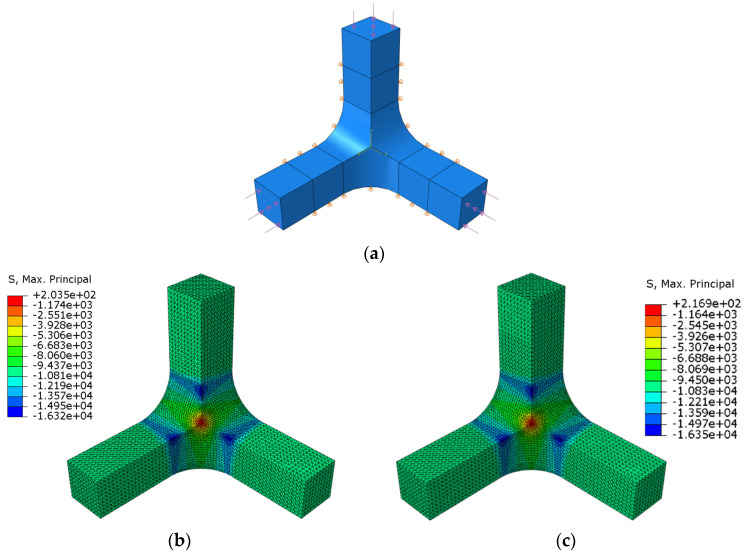
In geometry A for the CCC loading case: (**a**) Boundary conditions imposed by the fixture; (**b**) maximum principal stresses with ideal boundary conditions; and (**c**) maximum principal stresses with the boundary conditions imposed by the fixture.

The fixture shown in Section 2.2 is designed to constrain the specimen displacements, applying boundary conditions as close as possible to the symmetric ideal ones. The supporting system prevents the perpendicular displacements to the surfaces of the specimen that are in contact with the fixture up to a length of 30 mm from its centre (Figure 11a). The reason for this longitudinal restriction is to avoid the risk that the jaws could collide with the compression platens (Figure 4a–c). In the parametric analysis of the previous section, the numerical model considered the ideal symmetric boundary conditions. With comparison purposes, a stress-strain analysis is now performed with the boundary conditions imposed by the fixture in geometry A with *R* = 10 mm under a C-C-C loading case with an applied compressive stress of 10 kPa. The maximum principal stress fields are shown in Figure 11b for the case with ideal boundary conditions and in Figure 11c with the real ones. Stress concentration is produced at the join between the arms and the gauge region, specifically at the beginning of the rounding (in which the failure would be probably triggered). A 0.2% difference in the absolute value of the maximum stress is found, a disparity that is assumed to be negligible. Meanwhile, no remarkable difference is produced in the homogeneity of the stress fields along the diagonal path described in Figure 7a, so the fixture does not alter the stress distribution in the measurement area.

**Figure 12:**
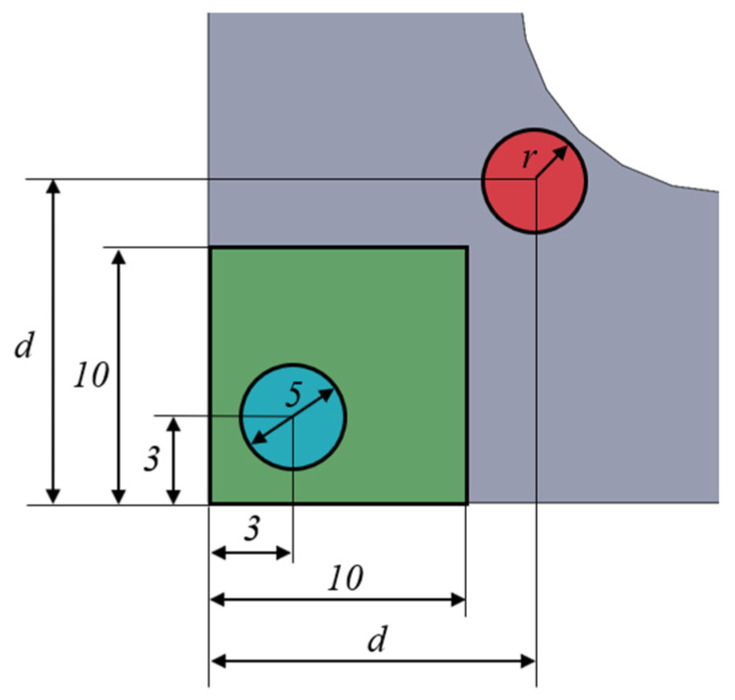
Measurement region on geometry A (green square), indicating the position and dimensions of the strain rosette (blue circle) and the opening (red circle). Units are in mm.

As well, the supporting system has to accomplish the main objective of letting measure the strain state of the triaxially loaded region during the tests. If a strain rosette is used, machined holes in the fixture are required for connecting its wires to the data acquisition system. The opening should have an influence on the measurable strains as low as possible. With this scope, a study of the homogeneity of the maximum principal stresses in the gauge region is carried out, comparing different hole diameters and their position on the fixture. In Figure 12, the squared area with a side of 10 mm that is marked in green over the specimen is the measuring zone. The blue circle represents the planned position of the strain rosette with 5 mm of diameter. It was decided that its centre should be located at 3 mm of the specimen edges, in order to assure that the 3 mm strain gauges are placed within a region in which the maximum principal stresses vary less than 10% of the central value (see Section 3.1.1). Meanwhile, the red circle symbolizes the opening that the fixing system should have for extracting the cables of the strain gauges. Its radius *r* and the centre position *d* are parametrized for reviewing its influence on the homogeneity of the stress fields. The opening radius varies between 2.0 mm ≤ *r* ≤ 3.5 mm and the hole centre varies between 2.5 mm ≤ *d* ≤ 10.0 mm, allowing for the perpendicular displacements of the specimen in the opened region. The analysis is developed applying a triaxial compressive stress of 10 kPa on geometry A with *R* = 10 mm and the real boundary conditions applied by the fixture. As a result, the maximum principal stresses are observed along the path η described in Figure 7a.

**Figure 13:**
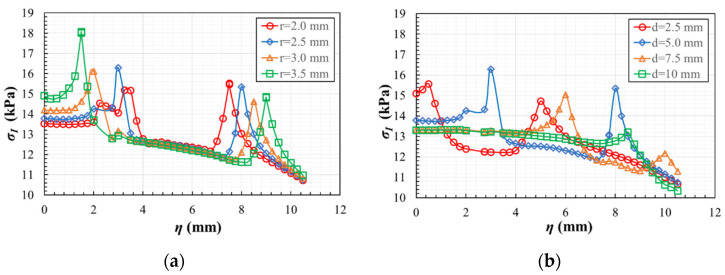
In geometry A for the CCC loading case: (**a**) influence of the opening radius for *d* = 5 mm; (**b**) influence of the centre position for *r* = 2.5 mm.

**Figure 14:**
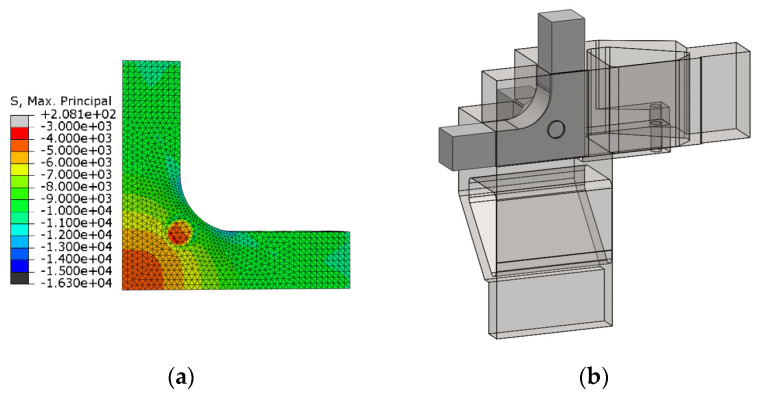
In geometry A for the CCC loading case: (**a**) Maximum principal stress field with an opening with *r* = 2.5 mm and *d* = 10 mm; (**b**) sketch of the fixture with a cut for visualizing the position and diameter of the opening.

Figure 13a depicts the influence of the opening radius for a fixed centre position *d* = 5 mm, while Figure 13b shows the influence of the centre position for a fixed radius *r* = 2.5 mm. From Figure 13a, it can be inferred that, for a fixed position of the opening, the larger the radius is, the more distorted get the stresses in the centre of the specimen. Therefore, it would be preferable to opt for a diameter as small as possible to facilitate the measurement of the strains in the area where the rosette is glued. From Figure 13b, the longer the distance d is, the less influence it has on the homogeneity of the maximum principal stress in the centre of the specimen. Therefore, it is desirable to set the centre of the opening as far as possible from the centre of the specimen. However, this distance is limited by the short length of the zone without plastic cover of the cables, which prevents the hole from being farther than 12.5 mm from the centre of the specimen. Taking into account both restrictive factors, an opening with a radius of 2.5 mm placed at a position of 10 mm with respect to the centre of the specimen is chosen, for which the effect of the opening in the maximum principal stress field is exhibited in Figure 14a. Figure 14b shows the representation of the fixture with the hole, in which a cut is made to visualize its position and size more clearly. Once the design is finished, although the specimen dimensions permit a correct application of the load, in order to ease the gripping of the specimen the length of the arms subjected to tensile loads could be increased from the original 40 mm to 60 mm.

## 4. Discussion

The methodology proposed for observing the triaxial response of the material, described in Section 2.2, can be better understood with a simulation of a test. Here the numerical results are discussed, although the future research should be focused on developing the tests experimentally.

**Figure 15:**
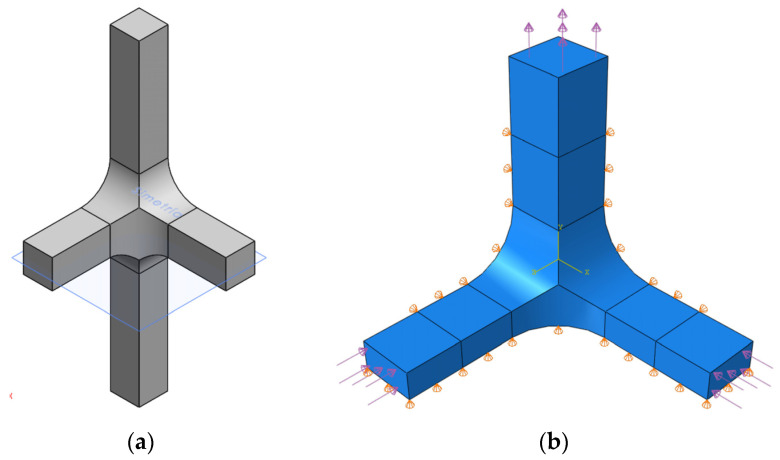
In geometry A for the CTC loading case: (**a**) Full configuration of the specimen; (**b**) geometry simulated with the real boundary conditions and applied stresses.

The response of geometry A for a case CTC under two different loading scenarios is selected to be studied due to its relatively complexity. In the loading scenario −50/50/−50, stresses of $\sigma_{ax}=-50$ MPa in the x-direction, $\sigma_{ay}=$50 MPa in the y-direction and $\sigma_{az}=-$50 MPa in the z-direction are applied in the arms of the specimens. Meanwhile, the stresses utilized in the loading scenario −50/25/−50 are $\sigma_{ax}=-$50 MPa in the x-direction, $\sigma_{ay}=$25 MPa in the y-direction and $\sigma_{az}=-$50 MPa in the z-direction. Figure 15a shows the specimen with its full configuration, but the simulation is developed studying one half of the geometry taking advantage of the symmetry (Figure 15b). The boundary conditions imposed by the fixture are taken into account.

**Figure 16:**
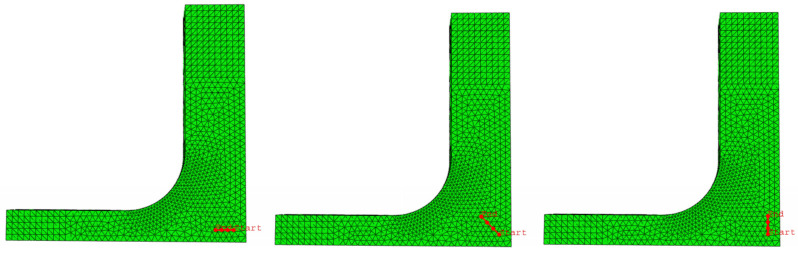
Paths at 0°, 45°, and 90° in geometry A for both loading scenarios.

Three paths of 3 mm length at 0°, 45°, and 90° (Figure 16) are established to simulate the three gauges that compose the rosette. For obtaining similar measurements as the strain gauges, an arithmetic mean of the strain values along each path is performed.

### 4.1. Geometry A for the CTC Case: Loading Scenario −50/50/−50

**Figure 17:**
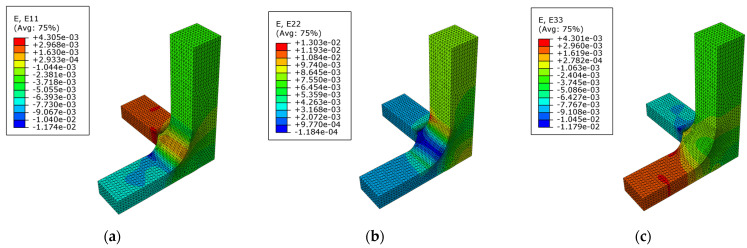
In geometry A for the CTC loading case and the scenario I: (**a**) Strain field in the x-direction; (**b**) strain field in the y-direction; (**c**) strain field in the z-direction.

The triaxial loading case $\sigma_{ax}=-50$ MPa, $\sigma_{ay}=$ 50 MPa, and $\sigma_{az}=-$50 MPa is reviewed, calculating the strain fields in the x-direction (Figure 17a), in the y-direction (Figure 17b), and in the z-direction (Figure 17c). The highest level of strain is produced in the arms of the specimens, appearing as the critical value in the tensed arm.

Applying the methodology described in Section 2.3, the strain tensor obtained from the averaged strains along the gauges is shown in Equation (5). The shear component *ε_xz_* cannot be determined from the direct normal measurements in the 0°, 45°, and 90° directions, so in the stress tensor it has been indicated by means of the symbol *. Nevertheless, visualizing the shear strains from the simulations in the region of interest, they are one order of magnitude lower than the normal components, so the normal components can be considered as the principal strains.

(5) $$\varepsilon^{-50/50/-50}=\left[\begin{array}{ccc} -4.0 & 0.2 & *\\ 0.2 & 8.4 & -0.2\\ {}* & -0.2 & -4.0 \end{array}\right]\cdot 10^{-3}$$

It can be highlighted that, despite applying the same absolute value of the stresses, the y-direction loaded under tension presents a deformation higher than the compressed directions. This is due to the fact that the y-direction has double the cross-sectional area of the x- and z-directions (in addition to the Poisson effect). The expressions of the hydrostatic and deviatoric parts of the strain tensor are given by Equations (6) and (7), respectively.

(6) $$\varepsilon_{h}^{-50/50/-50}=\left[\begin{array}{ccc} 0.1 & 0 & 0\\ 0 & 0.1 & 0\\ 0 & 0 & 0.1 \end{array}\right]\cdot 10^{-3}$$

(7) $$\varepsilon_{d}^{-50/50/-50}=\left[\begin{array}{ccc} -4.1 & 0.2 & *\\ 0.2 & 8.3 & -0.2\\ {}* & -0.2 & -4.1 \end{array}\right]\cdot 10^{-3}$$

### 4.2. Geometry A for the CTC Case: Loading Scenario −50/25/−50

**Figure 18:**
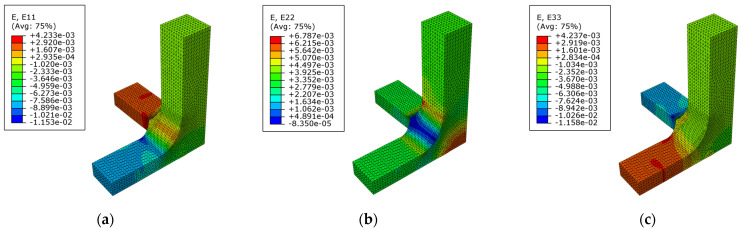
In geometry A for the CTC loading case and the scenario II: (**a**) Strain field in the x-direction; (**b**) strain field in the y-direction; (**c**) strain field in the z-direction.

Considering the scenario II with a loading case $\sigma_{ax}=-50$ MPa, $\sigma_{ay}=2$5 MPa, and $\sigma_{az}=-$50 MPa, the strain fields in the x-, y- and z-direction are depicted in Figure 18a–c, respectively. As in the previous case, the higher level of strain is produced out of the region triaxially loaded.

The estimation of the strain tensor from the direct measurements averaged along the length of each path is shown in Equation (8). As well, the hydrostatic and the deviatoric parts of the strain tensor are given in Equations (9) and (10), respectively.

(8) $$\varepsilon^{-50/25/-50}=\left[\begin{array}{ccc} -3.4 & 0.3 & *\\ 0.3 & 5.8 & 0.2\\ {}* & 0.2 & -3.4 \end{array}\right]\cdot 10^{-3}$$

(9) $$\varepsilon_{h}^{-50/25/-50}=\left[\begin{array}{ccc} -0.3 & 0 & 0\\ 0 & -0.3 & 0\\ 0 & 0 & -0.3 \end{array}\right]\cdot 10^{-3}$$

(10) $$\varepsilon_{d}^{-50/25/-50}=\left[\begin{array}{ccc} -3.1 & 0.3 & *\\ 0.3 & 6.2 & 0.2\\ {}* & 0.2 & -3.1 \end{array}\right]\cdot 10^{-3}$$

A higher hydrostatic component *p* is found under the loading scenario II. This leads to a lower deviatoric part, which in isotropic materials is responsible for failure. So, if the triaxial failure would be searched, this loading case would fail later in the region of interest. Nevertheless, in all of the cases the stress concentration is achieved out of the triaxially loaded region. So, the triaxial strength could not be accurately determined by the proposed tests, but they can be utilized for observing the triaxial response before failure. In the loading scenario I, the similar uniaxial stresses developed in the arms could let observe the triaxial response during a higher range of applied forces.

## 5. Conclusions

This work is centred on the design of a triaxial test adapted to the singular triaxial installation of the UCLM. Preceding studies have extrapolated the geometry of a biaxial cruciform specimen for triaxial testing of metals. Nevertheless, none of the previous specimens contemplated the possibility of direct strain measurement in the region of interest during testing. Therefore, the proposed geometries seek to leave free zones for measuring the strains in the triaxially loaded region, avoiding (as far as possible) the full geometry with six arms. Taking into account a polymer-based composite with apparent isotropic response in the three loading directions, the definition of a triaxial specimen is developed based on the results of FEM simulations. The final geometry, which fulfils the general dimensional constrains imposed by the installation and the requirements of stress homogeneity, is specimen A with a fillet radius between the arms and the gauge region of 10 mm. Nevertheless, it presents an unavoidable stress concentration out of the region of interest. Besides, a supporting system is proposed to constrain the displacements in the free surfaces of the specimen, imposing boundary conditions in the experiment which are as similar as possible to the symmetric ideal ones. As a first approximation in the design of the triaxial tests, the use of rosette gauges is proposed to acquire the experimental strains due to their simple use, the variety of sizes adaptable to the region of interest, and relatively low-cost. Other techniques could be utilised such as embedded fibre optical sensors or full-field DIC strain measurements. Discarding the utilization of inserted sensors since they could complicate the fabrication process of the specimen, the option of using DIC is feasible for future developments. In this case, two main lines of action should be followed: either to perform a new design process that considers an opening of the fixture that lets observe a zone large enough for the appropriate DIC observations, or to manufacture the proposed fixture with a transparent material (for example, acrylic (PMMA)), which could let visualize the triaxially loaded region.

As well, the methodology used to determine the full strain tensor in the triaxially loaded region from the experiments is described analytically and with the help of FEM virtual testing. The strain gauges are proposed to be placed within a region in which the measurements vary less than 10% of the central value, so the experimentally determined strain tensor is considered as representative of the strain state in all of the region of interest. In particular, in this work the deviatoric part of the strain tensor is used to estimate the susceptibility of the polymer-based composite to failure due to the imposed triaxial strain state. Thus, loading cases that generate higher deviatoric components of the strain tensor are considered more likely to damage the region of interest. However, experimental failure is expected to occur in the arms of the specimens which are uniaxially loaded, since in all reviewed scenarios the numerical results show higher levels of stress concentration outside the triaxially loaded region. Therefore, the triaxial strength could not be accurately determined by the proposed tests. However, they can be used to examine the triaxial response before failure. The triaxial response of the material could be observed over a larger range of applied forces if similar uniaxial stresses are developed in the arms of the sample.

## Figures and Tables

**Table 1:** In geometry A and a fillet radius *R* between 0.5 mm and 28.0 mm: η-coordinate at which the maximum principal stress along the path takes the values of 1.05 *σ*_0_ and 1.1 *σ*_0_.

***R*** **(mm)**	0.5	1.0	2.0	4.0	6.0	8.0	10.0	12.0	14.0	16.0	18.0	20.0	22.0	24.0	26.0	28.0
**1.05 *σ*_0_ at** η **-coordinate (mm)**	5.1	4.6	4.2	3.5	3.0	2.9	2.9	2.7	2.4	2.4	2.3	2.8	2.8	2.9	2.8	2.9
**1.1 *σ*_0_ at** η **-coordinate (mm)**	5.9	5.7	5.5	5.2	4.7	4.7	4.2	4.2	4.0	3.9	3.9	3.8	3.8	3.7	3.8	3.8

**Table 2:** In geometry B with an external radio *R_e_* = 1 mm and internal radius *R_i_* between 1.1 mm and 2.9 mm: η-coordinate at which the maximum principal stress along the path takes the values of 1.05 *σ*_0_ and 1.1 *σ*_0_.

***R_i_* (mm)**	1.1	1.2	1.3	1.4	1.5	1.6	1.7	1.8	1.9	2.0	2.1	2.2	2.3	2.4	2.5	2.6	2.7	2.8	2.9
**1.05 *σ*_0_ at** η **-coordinate (mm)**	6.2	6.1	5.7	5.6	5.6	5.6	5.5	5.3	5.0	4.8	4.8	4.7	4.5	4.4	4.3	4.1	4.0	4.0	3.9
**1.1 *σ*_0_ at** η **-coordinate (mm)**	6.7	6.5	6.4	6.4	6.5	6.2	6.1	5.9	5.8	5.6	5.5	5.5	5.5	5.3	5.1	4.8	4.8	4.7	4.8

## Data Availability

Not applicable.
